# An Integrated Computational and Experimental Approach to Identifying Inhibitors for SARS-CoV-2 3CL Protease

**DOI:** 10.3389/fmolb.2021.661424

**Published:** 2021-05-17

**Authors:** Tianhua Zhai, Fangyuan Zhang, Shozeb Haider, Daniel Kraut, Zuyi Huang

**Affiliations:** ^1^Department of Chemical and Biological Engineering, Villanova University, Villanova, PA, United States; ^2^School of Pharmacy, University College London (UCL), London, United Kingdom; ^3^Department of Chemistry, Villanova University, Villanova, PA, United States

**Keywords:** SARS-CoV-2, 3CL protease inhibitors, docking, QSAR, IC50

## Abstract

The newly evolved SARS-CoV-2 has caused the COVID-19 pandemic, and the SARS-CoV-2 main protease 3CLpro is essential for the rapid replication of the virus. Inhibiting this protease may open an alternative avenue toward therapeutic intervention. In this work, a computational docking approach was developed to identify potential small-molecule inhibitors for SARS-CoV-2 3CLpro. Totally 288 potential hits were identified from a half-million bioactive chemicals via a protein-ligand docking protocol. To further evaluate the docking results, a quantitative structure activity relationship (QSAR) model of 3CLpro inhibitors was developed based on existing small molecule inhibitors of the 3CLpro^SARS– CoV– 1^ and their corresponding IC_50_ data. The QSAR model assesses the physicochemical properties of identified compounds and estimates their inhibitory effects on 3CLpro^SARS– CoV– 2^. Seventy-one potential inhibitors of 3CLpro were selected through these computational approaches and further evaluated via an enzyme activity assay. The results show that two chemicals, i.e., 5-((1-([1,1′-biphenyl]-4-yl)-2,5-dimethyl-1H-pyrrol-3-yl)methylene)pyrimidine-2,4,6(1H,3H,5H)-trione and N-(4-((3-(4-chlorophenylsulfonamido)quinoxalin-2-yl)amino)phenyl)acetamide, effectively inhibited 3CLpro SARS-CoV-2 with IC_50_’s of 19 ± 3 μM and 38 ± 3 μM, respectively. The compounds contain two basic structures, pyrimidinetrione and quinoxaline, which were newly found in 3CLpro inhibitor structures and are of high interest for lead optimization. The findings from this work, such as 3CLpro inhibitor candidates and the QSAR model, will be helpful to accelerate the discovery of inhibitors for related coronaviruses that may carry proteases with similar structures to SARS-CoV-2 3CLpro.

## Introduction

Coronaviruses are enveloped RNA viruses that infect the respiratory tracts of humans and animals (De Wilde et al., 2018; Huang et al., 2020). Severe acute respiratory syndrome (SARS) and Middle East Respiratory Syndrome (MERS) are coronaviruses that have caused many human deaths in the twenty-first century (Hilgenfeld and Peiris, 2013). A new coronavirus, named SARS-CoV-2, was detected in December 2019 in Wuhan, China (Kong et al., 2020). It was then quickly traced in other countries (Lai et al., 2020). The disease caused by SARS-CoV-2, i.e., COVID-19, is a severe health problem, not only because of its rapid spread worldwide, but also due to its high fatality rate (Lai et al., 2020; Xu et al., 2020). In particular, COVID-19 has caused more than 1.82 million deaths worldwide as of December 31st, 2020 (CSSE, 2020). Effective vaccines and anti-viral treatments are immediately needed.

Although vaccines are the most efficient way to end the COVID-19 pandemic and a couple of vaccines have been authorized for emergency use to control the pandemic, safety issues for certain people, e.g., those with allergies, those who are pregnant, and those with immune disorders, are still concerned (CDC, 2021b). 3CLpro inhibitors could be a potential therapeutic for infected patients, especially as sufficient vaccination to reach herd immunity will take some time (Dhama et al., 2020). We must therefore in parallel develop therapeutic drugs for those infected with coronavirus. Furthermore, we have observed SARS-COV-2 variants spread (CDC, 2021a). Though so far there is no indication that known mutations will prevent vaccines from being effective, it remains a possibility. Exploring effective anti-COVID-19 agents, that might also be useful for future coronavirus variants, is valuable. The structure of the main SARS-CoV-2 protease, 3CLpro, is highly conserved across coronavirus variants (Abian et al., 2020). Identifying therapeutic agents to inhibit 3CLpro might then be useful for the treatment of COVID-19 patients, and these agents will remain valuable for the treatment of infections caused by mutated SARS-CoV-2 in the future (Anand et al., 2003; Klemm et al., 2020). Thus, it is crucial that we continue to develop new anti-viral therapies, and *in silico* screening and experiment validation can be an important first step in this process.

One of the efficient ways to identify effective anti-COVID-19 agents is revisiting existing drugs that have been previously approved for treating other viral infections. However, the efficacies of those tried so far are not as high as expected. For example, although Remdesivir, which was approved for Ebola virus, was able to shorten the recovery time and decrease the mortality rate of COVID-19 (Beigel et al., 2020), various side effects were reported (Grein et al., 2020). Another anti-COVID-19 clinical study was based upon a combination of the HIV 3CLpro inhibitors lopinavir and ritonavir. Lopinavir, which acts against the 3-Chymotrypsin like protease (3CLpro) associated with HIV, is not a particularly potent therapeutic agent against SARS-CoV-2. The concentration necessary to inhibit viral replication is relatively high as compared with the serum levels found in patients treated with lopinavir–ritonavir. It is thus not surprising to find that no benefit was observed with lopinavir–ritonavir treatment when compared to the standard care protocol (Li and De Clercq, 2020). Therefore, effective anti-COVID-19 agents still need to be developed.

Understanding how SARS-CoV-2 invades the human body is helpful for the development of effective anti-COVID-19 agents. The SARS-CoV-2 attacks the lower respiratory system and the gastrointestinal system (Liu C. et al., 2020). Before entering host cells, the spike (S) protein on coronavirus binds to the angiotensin converting enzyme 2 (ACE2) on the cell surface (Perlman and Netland, 2009). After the viral RNA enters the host cell, the replication of viral RNA occurs in double membrane vesicles (DMV) (Stertz et al., 2007; Perlman and Netland, 2009). The 3 chymotrypsin-like protease (3CLpro) is essential for viral replication. 3CLpro cleaves the transcript polyprotein, releasing both itself and other functional proteins (Anand et al., 2003; Ratia et al., 2008). Inhibitors of 3CLpro^SARS– CoV– 1^ have been extensively studied (Liu Y. et al., 2020). This motivates us to make use of existing inhibitors of 3CLpro^SARS– CoV– 1^ to accelerate the identification of effective inhibitors of 3CLpro^SARS– CoV– 2^ to combat COVID-19.

Coronavirus 3CLpro is enzymatically active as a homodimer. Its monomeric subunit is irreversibly inactivated, as its catalytic machinery is frozen in the collapsed state, characterized by the formation of a short 3_10_-helix from an active-site loop (Shi et al., 2008). Inhibiting dimerization of the 3CLpro monomer is thus one way to inhibit 3CLpro. However, dimerization inhibitors typically target the dimerization interface and thus compete with the attractive forces between subunits (Barrila et al., 2006). A previous study suggested covalent inhibitors of 3CLpro targeting the nucleophilic cysteine 145 in the active site eliminated the enzyme activity (Pillaiyar et al., 2016). Additionally, a cluster of serine residues (Ser139, Ser144, and Ser147) was identified near the active site cavity and was susceptible to being targeted by compounds containing boronic acid compounds, which are particularly effective inhibitors, with *K*_i_’s as strong as 40 nM (Bacha et al., 2004). Targeting the active site is thus preferred for 3CLpro inhibition.

Attempts have been made to provide a complete description of the structural features and detailed mechanisms of action of existing 3CLpro^SARS– CoV– 1^ inhibitors. Many peptide inhibitors were designed to mimic natural viral polypeptides and covalently bind to the active site Cys_145_. Despite their potent inhibition of 3CLpro^SARS– CoV– 1^ and relatively long half-life in buffer at neutral pH values, these peptide inhibitors are likely to be problematic, because of their high propensity to be rapidly hydrolyzed by lipases, esterases, and other enzymes in mammalian cells. Moreover, these compounds can potentially react nonspecifically with other thiols or nucleophiles in mammalian cells, thereby leading to toxicity (Pillaiyar et al., 2016). The other category of 3CLpro inhibitors includes noncovalent or reversible covalent inhibitors, which have advantages regarding side effects and toxicity. These inhibitors were discovered by high throughput screening of synthetic compounds and natural products, such as etacrynic acid derivatives, isatins, flavonoid derivatives, terpenoids, active heterocyclic ester analogs, pyrazolones and pyrimidines (Jacobs et al., 2013; Turlington et al., 2013; Liu Y. et al., 2020).

On the basis of the aforementioned 3CLpro^SARS– CoV– 1^ inhibitors and IC_50_ data, we implemented a protein-ligand docking approach (refer to Zhang F. et al., 2020 as an example) to identify potential 3CLpro inhibitors and then developed a quantitative structure–activity relationship (QSAR) model to narrow down candidates (i.e., with low IC_50_) for 3CLpro^SARS– CoV– 2^. A three-dimensional QSAR model attempts to correlate 3D molecular structure to biological activity, often using a variety of molecular descriptors such as physicochemical, topological, electronic and steric properties (Nantasenamat et al., 2009). In particular, 3D Atomic Property Fields (APF) QSAR methods developed by ICM calculate physico-chemical properties of superimposed chemicals and utilize their half-inhibition data to weight contributions for each property through Partial-Least-Squares (PLS) regression modeling (Totrov, 2008). Such a QSAR model allows for the quantitative prediction of pharmacological activities of congeneric unknown compounds so that it can be used to direct the design of novel derivatives with enhanced activity (Totrov, 2008). While hundreds of compounds were screened by their binding affinity to the 3CLpro through automated molecular docking (Sirois et al., 2004; Achilonu et al., 2020), the resulting docking scores had a limited ability to accurately predict inhibitor efficacy (Kitchen et al., 2004). It is thus necessary to further implement the 3D QSAR model to evaluate physicochemical properties and potential inhibitory effectiveness of those compounds identified through the molecular docking. In particular, the 3D QSAR model is able to predict IC_50_’s of those compounds with high-binding scores. The inhibitors with good predicted IC_50_ values are good candidates for further experimental validation. One hypothesis underlying our work is that the 3D QSAR model that links the structures of 3CLpro^SARS– CoV– 1^ inhibitors to IC_50_ values can be applied to identify inhibitors of 3CLpro^SARS– CoV– 2^. The rationale behind this hypothesis is that the crystal structure of 3CLpro^SARS– CoV– 2^ is similar to that of 3CLpro^SARS– CoV– 1^ and the active pockets are conserved between these two 3CL proteases (Liu Y. et al., 2020). Furthermore, some aldehyde and α-ketoamide compounds serve as broad-spectrum inhibitors of 3CLpro from both SARS-CoV-1 and SARS-CoV-2 (Zhang L. et al., 2020).

## Materials and Methods

**FIGURE 1:**
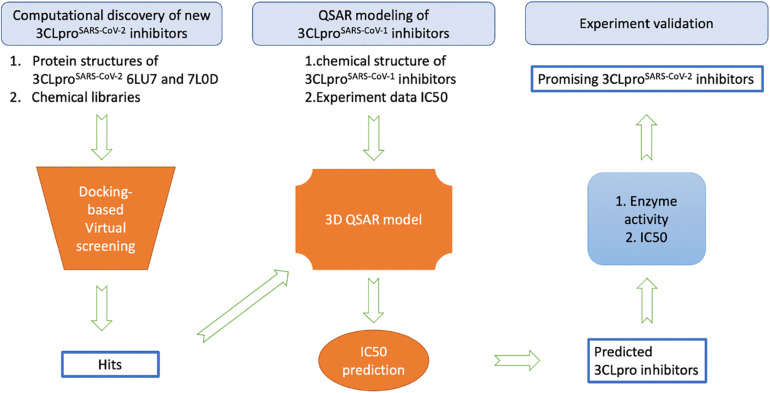
The overview of the proposed workflow to discover new inhibitors of 3CLpro^SARS– CoV– 2^.

While the detailed methods are introduced in each of the following subsections, Figure 1 provides an overview of the proposed workflow for identifying inhibitors of SARS-CoV-2 3CLpro: (1) FDA-approved drugs and IBScreen compounds libraries were docked into the crystal structure of SARS-CoV-2 3CLpro (PDB ID 6LU7) to identify strong binders using the docking program ICM; (2) Half-inhibition concentration (IC_50_) data along with the structures of existing 3CLpro^SARS– CoV– 1^ inhibitors were used to develop a QSAR model to predict the IC_50_ of the new 3CLpro^SARS– CoV– 2^ inhibitors using ICM (Jacobs et al., 2013; Turlington et al., 2013; Pillaiyar et al., 2016); (3) the top inhibitor candidates (lowest docking score and predicted IC_50_ values) were tested in an enzyme activity assay at the 100 μM concentration; (4) the inhibitor candidates with the best performance in the initial enzyme activity assay were tested in the IC_50_ experiment. For the QSAR modeling, 50% of compounds in the literature dataset were used for training the QSAR model, while the other compounds were reserved to validate the model.

### Docking-Based Virtual Screening

Structures of 3CLpro^SARS– CoV– 2^ (PDB ID 6LU7) bound with inhibitors were obtained from the Protein Data Bank (PDB) (Jin et al., 2020). Since 3CLpro^SARS– CoV– 2^ structure 6LU7 was the first one published and it was well-studied, the 6LU7 structure was used for initial virtual screening. The structure 6LU7 was first converted into the format used in ICM and then modified by removing ligand, deleting water, and adding hydrogens. The following residues were further optimized: three protonation states and two rotations of all histidine (His) residues and 180-degree flip of asparagine (Asn) and glutamine(Gln) residues were tried to minimize the global energy. Particularly, both His41 and His163 at the active site were in Nδ1-protonated π tautomer state. The ligand binding pocket was predicted by icmPocketFinder with a recommended tolerance level 4.6 by ICM. The largest pocket covering the crystalized ligands was selected. The docking grid was generated with a size of 27.6 × 18.0 × 24.5 Å and the probe was placed at the center of the box, shown in Supplementary Figure 1. Crystalized ligand N3 from the structure 6LU7 was extracted and redocked into the receptor, generating scores of △G = −29.02 kcal/mol. The docking conformation gave an RMSD of 0.6 Å relative to the original, but it does not contain a covalent bond with Cys145 (Supplementary Figure 2). Such a non-covalent docking mode could mimic the N3 inhibitor binding at the active site prior to the covalent reaction. Therefore, the docking score is meaningful as a threshold for virtual screening. In terms of the chemical libraries to screen, FDA approved drugs (2,305 compounds) (Patridge et al., 2016) provided the first set, while additional compounds came from InterBioScreen’s high-quality compound database (>550,000 compounds) for drug screening (Roy et al., 2015). The InterBioScreen database was preferred in this work as it has been widely implemented for repurposing, high-throughput screening, and hit identification. Compounds were first filtered by “Lipinski’s rules of five” (Lipinski et al., 1997) and around 0.45 million compounds were maintained and docked into the structure 6LU7. The virtual screening was conducted using scoring function 2005 and docking effort 1. Docked compounds with scores lower than those of ligand 6LU7 (−29.02 kcal/mol) were retained. We validated docking conformation for each potential hit by redocking it into a second structure 7L0D and evaluated the RMSD values (Supplementary Figure 3). The ICM score was calculated as a binding free energy (△G) that is composed of the hydrogen bond energy, hydrophobic energy in exposing a surface to water, van der Waals interaction energy, internal conformational energy of the ligand, desolvation of exposed h-bond donors and acceptors, solvation electrostatics energy change upon binding, loss of entropy, and the potential of mean force score (Neves et al., 2012). Additionally, LogP, log of the octanol/water ratio, was calculated by ICM to allow evaluation of the water solubility and bioavailability of each drug.

### 3D QSAR Analysis

QSAR analysis consists of two steps: the first step deals with the generation of a QSAR model based on known 3CLpro^SARS– CoV– 1^ inhibitors, while the second step is focused on the prediction of inhibitory activity of new compounds (Totrov, 2008). The activity data for the non-covalent inhibitors of 3CLpro^SARS– CoV– 1^, including decahydroisoquinoline derivatives, octahydro-isochromene derivatives, pyrazolone and pyrimidines, compounds with 3-pyridyl or triazole or piperidine moiety and natural product derivatives, were obtained from the literatures (Jacobs et al., 2013; Turlington et al., 2013; Pillaiyar et al., 2016). 3D structures of the inhibitors were converted from SMILES based on Merck Molecular Force Field (MMFF) atom type and force field optimization. A 3CLpro inhibitor ML188 occupies all four sub-pockets in the active site (PDB ID 3V3M), which might include most of binding modes (Jacobs et al., 2013). Therefore, the ML188 was used as a template for 3D alignment as the ligand. In total, 65 inhibitors were aligned to the template using the flexible APF superposition method (Totrov, 2008). Subsequently, 35 compounds were used as the training set to build a 3D QSAR model and 30 compounds were grouped as the testing set for validation.

For each of the aligned compounds, seven physicochemical properties were calculated and pooled together by APF. The APF method, designed by ICM, uses the assignment of a 3D pharmacophore potential on a continuously distributed grid using physio-chemical properties of the selected compound(s) to classify or superimpose compounds (Totrov, 2011). These properties include hydrogen bond donors, acceptors, carbon hybridization, lipophilicity, size, electropositivity/negativity and charges. Based on the half-inhibition data obtained from the literature and the 3D aligned structures for the known compounds, weighted contributions for each APF component were obtained to allow quantitative activity predictions for unknown compounds. The optimal weight distributions were assigned by partial least-squares (PLS) methodology, where the optimal number of latent vectors for PLS was established by leave-one-out cross-validation on the training set. Then the weighted contributions were added together (Totrov, 2008). All potential 3CLpro inhibitors (i.e., those that had ΔG < −29.02 from the docking experiment) were subjected to the conversion and alignment protocol using ICM. Finally, the top 71 compounds were selected for further experimental validation.

### Enzyme Activity Assay and IC_50_

#### Potential Inhibitors Tested and Stock Solution Preparation

71 potential inhibitors were tested. Among them, 70 compounds (listed in Supplementary Table 2) were purchased from InterBioScreen Itd. (IBS, Russia). The remaining potential inhibitor was pentagastrin, which is an FDA-approved drug (MedChemExpress Inc., NJ). Compounds were dissolved in DMSO (Sigma-Aldrich Inc., St. Louis, MO) to reach a final concentration of 10 g/L. The stock solutions were stored at −20°C until further use.

#### Enzyme Activity Assay

In each experimental group, 30 μL of 15 nM purified recombinant 3CLpro (BPS Bioscience Inc., CA) and 10 μL of 500 μM prepared inhibitor solution in 5% aqueous DMSO was added into a black 96-well plate (Nunc U96). 30 μL of 15 nM purified recombinant 3CL-pro and 10 μL of 500 μM GC-376 (a known inhibitor) were added as an inhibitor control (Fu et al., 2020). 30 μL of 15 nM purified recombinant 3CL-pro and 10 μL of 5% DMSO in water were added as a positive control. After preincubation at room temperature with slow shaking for 30 min, 10 μL of 200 μM substrate solution DABCYL-KTSAVLQSGFRKME-EDANS (BPS Bioscience Inc., CA) was added into each well. The final concentration of tested compounds and the inhibitor control were 100 μM. The plate was incubated at 25°C with slow shaking for 2 h, and at the same time the fluorescence was measured every 3 min at an excitation wavelength of 360 nm and an emission wavelength of 460 nm on a CLARIOstar Plus plate-reader (BMG Labtech, Germany). Duplicate experiments were performed and the enzyme activity in the inhibitor control was used to select effective inhibitors of 3CL pro.

#### IC_50_ Test

The top two inhibitors were selected from the enzyme activity test for the IC_50_ test. A similar procedure as used in the enzyme activity test was implemented in the IC_50_ test, except that the compound final concentration was varied from 200 to 6.25 μM by two-fold serial dilution (Balouiri et al., 2016). Experiments were performed in triplicate. 3CLpro and each compound were incubated at 25°C with slow shaking for 2 h, the emission fluorescence was detected every 3 min. Enzyme activity was determined as the slope of florescence vs. time. Relative enzyme activities were calculated as the ratio of enzyme activities for the compound-treated groups to the positive control (i.e., no inhibitor). The IC_50_ values were determined by fitting the relative enzyme activity as a function of compound concentration to the following Hill equation using Graphpad (version 9.1.0).

(1) $$y=\frac{100}{1+{\left(\frac{I\,C_{50}}{x}\right)}^{n}}$$

where *y* is the relative enzyme activity, *x* is the compound concentration, and *n* is the Hill slope.

## Results

### Identification of Potential SARS-CoV-2 3CLpro Inhibitors

After screening half million compounds, 288 hits in total were identified from the FDA-approved compound library and the IBScreen database. Docking scores were used to estimate ligand binding affinity for 3CLpro, and the results are shown in Supplementary Table 3. Potential inhibitors were defined as those that were predicted to bind more tightly (lower scores) than the crystallographic ligands. ΔG (binding to 6LU7) of the predicted strong binders ranged from −41.3 to −30 kcal/mol, with a cutoff of −29.02 kcal/mol. The lower docking scores indicated relatively higher binding affinity and stronger ligand-receptor interaction. The compounds were all predicted to be bound within the active site of 3CLpro in a position similar to the crystallographic ligands. After the first run of virtual screening was finished, more 3CLpro^SARS– CoV– 2^ structures, including structures with non-covalent binders (e.g., 7L0D), became available. Although structures 6LU7 and 7L0D are identical in sequence and similar in secondary structure, some residues around the active site are not identical in conformation. These residues, e.g., T25, M49, M165, and P168, may slightly change the docking grid. Therefore, we redocked the hits identified on the basis of structure 6LU7 into structure 7L0D to further validate the ligand conformations. It turned out that compounds showed similar docking conformations (RMSD < 2Å) between the two structures. The results are provided in Supplementary Table 3. This agreement between the structures indicates that the presented approach should be applicable to other 3CLpro^SARS– CoV– 2^ structures.

**FIGURE 2:**
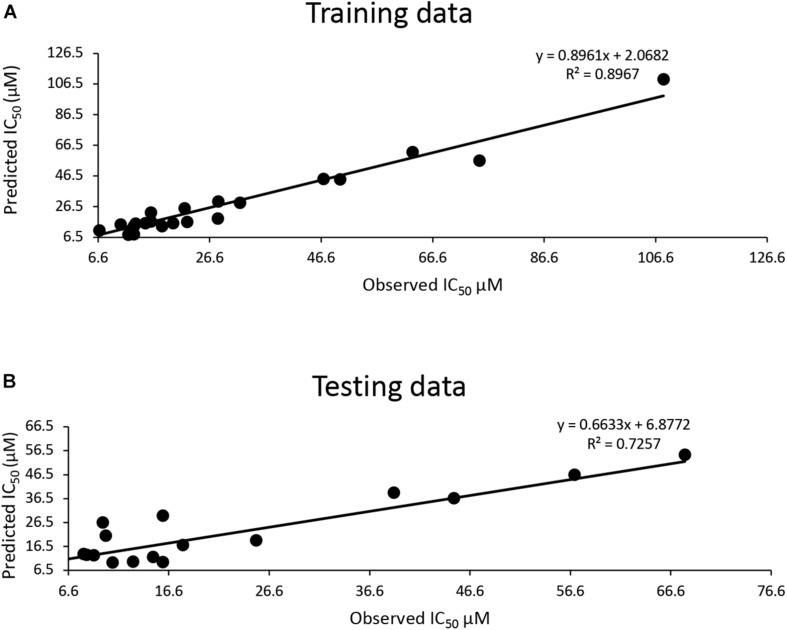
Development of a QSAR model: **(A)** The QSAR model generated by the training data suggests a good fit (*R*^2^ = 0.9); **(B)** a strong correlation (*R*^2^ = 0.72) between actual IC_50_ μM and predicted IC_50_ μM for the test data.

The training dataset for the QSAR model (35 known 3CLpro^SARS– CoV– 1^ inhibitors) had a good quality fit (*R*^2^ = 0.8967) (Figure 2A and Supplementary Table 3), while the testing dataset suggested the predicted IC_50_ was still correlated to the actual IC_50_ (*R*^2^ = 0.7257) for 30 additional known inhibitors that hadn’t been used in training (Figure 2B and Supplementary Table 3). The QSAR model generated using these 3CLpro^SARS– CoV– 1^ inhibitors was then used to evaluate the IC_50_ of potential 3CLpro^SARS– CoV– 2^ inhibitors. The 288 identified hits were input into the developed QASR model to estimate half-inhibition values. The predicted IC_50_ for each compound were ranged from 0.35 to 46.7 μM. The top 71 compounds with predicted IC_50_’s ranging from 0.35 to 19.86 μM (Supplementary Table 3), were selected for further evaluation in an enzyme activity assay.

### Inhibitory Activity and IC_50_ of the Selected Compounds

**FIGURE 3:**
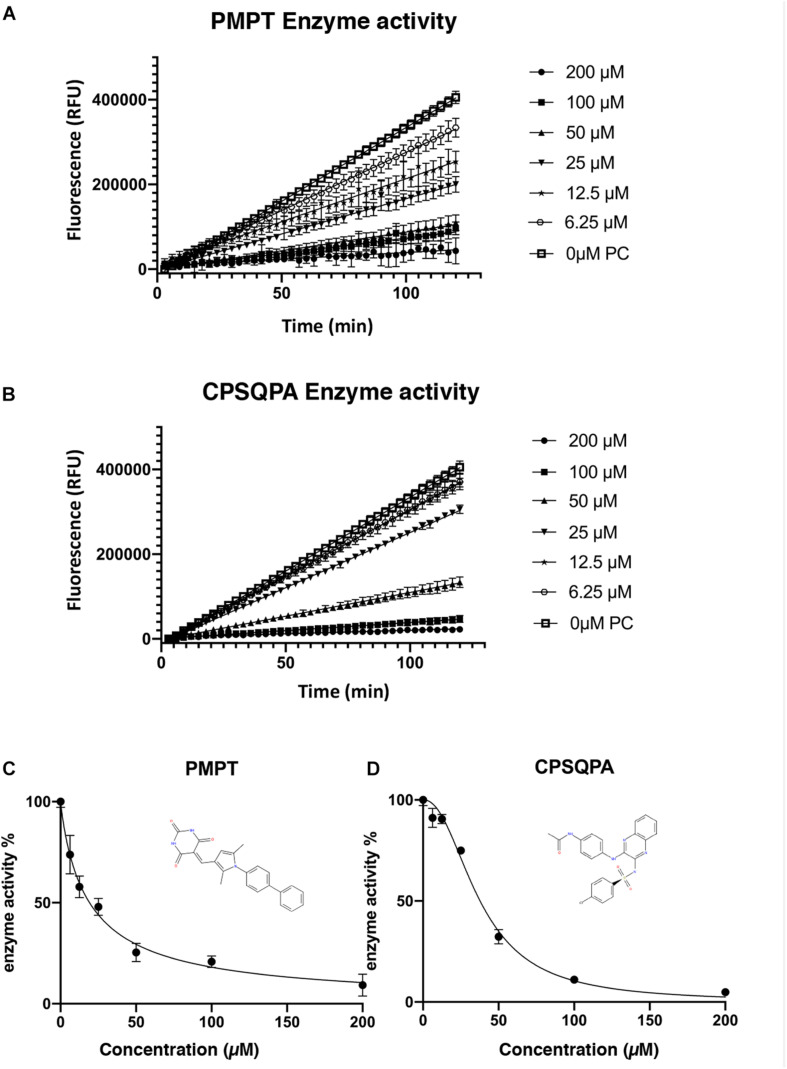
Enzyme activities **(A,B)** and IC_50_ curve **(C,D)** of newly identified inhibitors, PMPT and CPSQPA. 3CLpro and inhibitors were incubated at 25°C with slow shaking for 2 h, with fluorescence recorded every 3 min **(A,B)**. The slope of each curve corresponded to the enzyme activity. Relative enzyme activities were calculated as ratio of compound-treated groups and positive control (no inhibitor) to determine IC_50_. Data analysis and curve fitting were conducted using the program Graphpad.

Before testing the IC_50_ value of the predicted inhibitors, we did a preliminary screening of the 71 lead compounds identified from the docking and QSAR modeling. For this purpose, *in vitro* fluorescence resonance energy transfer (FRET) enzymatic assays were conducted in the presence of 10 nM enzyme and 100 μM of each inhibitor. 33 compounds were not soluble in water (5% DMSO, room temperature) at a concentration of 100 μM. There were 29 additional soluble compounds that showed no inhibition. Nine small-molecule compounds were found to have an inhibitory effect in the enzyme activity assay (Supplementary Table 2). Compounds listed in Table 1, 5-((1-([1,1′-biphenyl]-4-yl)-2,5-dimethyl-1H-pyrrol-3-yl)methylene)pyrimidine-2,4,6 (1H,3H,5H)-trione (abbreviated as PMPT), and N-(4-((3-(4-chlorophenylsulfonamido)quinoxalin-2-yl) amino) phenyl)acetamide (abbreviated as CPSQPA), which were among the highest scoring soluble compounds in the QSAR screen, appeared to have the highest inhibition potential in the preliminary screen. PMPT and CPSQPA at a concentration of 100 μM reduced the activities of 3CLpro to 21 and 11%, respectively. 100 μM Pentagastrin, an FDA approved drug, reduced 3CLpro’s activity to 31% (Supplementary Table 2). GC376, a known covalent 3CLpro inhibitor with its IC_50_ as 0.15 μM (Fu et al., 2020), suppressed initial activity to 5% (Supplementary Table 2). We therefore proceeded to measure the IC_50_ for CPSQPA and PMPT. Enzyme activities in the presence of the two compounds (concentration gradients from 200 to 6.25 μM) were plotted in Figures 3A,B and listed in Supplementary Table 1, and IC_50_ curves are shown in Figures 3C,D. The IC_50_ of PMPT was determined to be 19 ± 3 μM by nonlinear regression of the rate of enzyme activity as a function of inhibitor concentration (*R*^2^ = 0.97). The IC_50_ of CPSQPA was 38 ± 3 μM as calculated by same fitting method (*R*^2^ = 0.99).

**TABLE 1:** Newly discovered SARS-CoV-2 3CLprotease inhibitors.

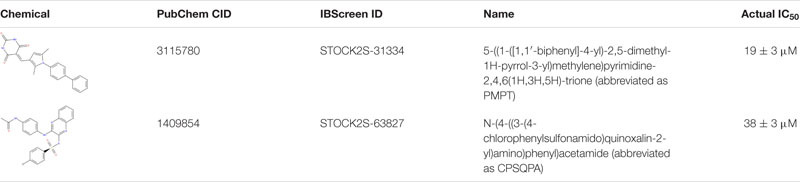

### Binding Conformations of Compounds Predicted by Docking

**FIGURE 4:**
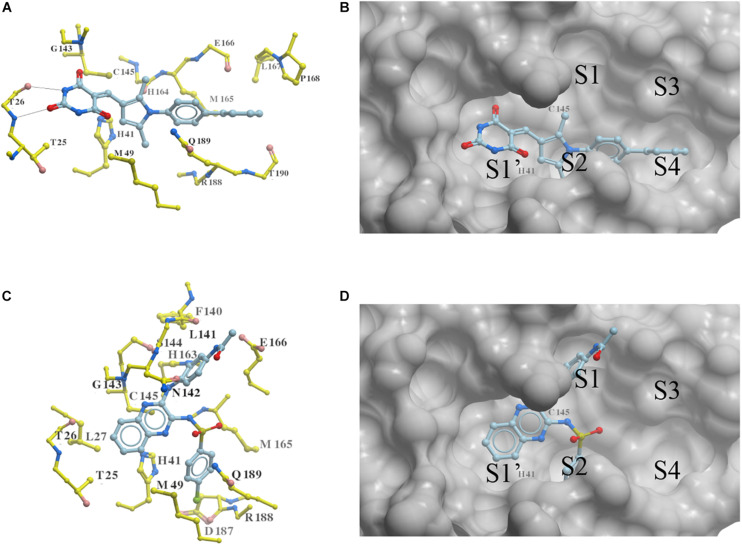
Docking conformations of newly identified 3CLpro^SARS– CoV– 2^ inhibitors: **(A,B)** PMPT; **(C,D)** CPSQPA. 3CLpro was modified from the 6LU7 structure by removing the ligand and water. Protein is colored in yellow. Compounds are colored in sky blue. Amino acid residues interacting with the ligands are labeled. Surface of the protein is displayed and colored in gray **(B,D)**. Hydrogen bonds between PMPT and 3CLpro are shown as black lines **(A)**. As CPSQPA likely has a net charge of -1 (sulfonamide p*K*_a_’s generally are in the range of 5.0–7.0), the anionic form was docked into the protein **(C,D)**. S1′, S1, S2, S3, and S4 are the sub-pockets for binding.

Two new 3CLpro^SARS– CoV– 2^ inhibitors PMPT and CPSQPA were predicted to bind at the active site of the protease. Docking conformers and ligand-receptor interactions (to structure 6LU7) are presented in Figure 4 for the purpose of illustration. PMPT and CPSQPA were predicted to non-covalently bind to the substrate binding site of 3CLpro^SARS– CoV– 2^ and competitively prevent the substrate from binding. Based on our docking model, PMPT forms two hydrogen bonds with residue Thr26 and interacts with other residues, such as Asn142, His164, Leu167 and Met165, via Van der Waals or hydrophobic interactions (Figure 4A and Supplementary Figure 4). It binds to sub-pockets S1′, S2, and S4, and thereby blocks the active site His41 and Cys145 (Figure 4B). CPSQPA, in a “−1” anionic form, binds to the S1′, S1, and S2 pockets and interacts with additional residues (Figures 4C,D and Supplementary Figure 5). Hydrogen bonds formed by PMPT could enhance the binding affinity, which may explain why PMPT has a lower IC_50._ In addition, both PMPT and CPSQPA showed similar docking poses in Structures 6LU7 and 7L0D, with RMSD’s of 0.68 and 0.86 Å, respectively (Supplementary Figures 5,6).

## Discussion

Molecular docking is a common approach to quickly identify potential 3CLpro inhibitors against SARS-CoV-2 in previous research (Jin et al., 2020). Although docking scores, to some degree, evaluate the binding affinity of the compound to the docking target, imperfections of scoring functions continue to be a major limiting factor impacting the accuracy of the docking prediction (Kitchen et al., 2004). To effectively identify novel 3CLpro^SARS– CoV– 2^ inhibitors, an integrated docking-based virtual screening and QSAR method was conducted in this work. ICM scores of the identified 288 compounds indicated that they might bind at the active site of 3CLpro with high affinity. The docking scores for the structure 6LU7 were used to narrow down the candidates for the next step in which IC_50_ values of the selected candidates were predicted by a QSAR model. In particular, the developed QSAR model gave a quantitative ligand-based virtual screening approach to further evaluate the physico-chemical properties of compounds and estimate their IC_50_ values on the basis of the data for the known inhibitors for 3CLpro^SARS– CoV– 1^ to narrow down the number of compounds for testing. Indeed, nine compounds showed an inhibitory effect among the 71 candidates tested, with a success rate of 12.7%. The two inhibitors found in this work, i.e., PMPT and CPSQPA, were ranked as top candidates in the virtual screen with insoluble compounds removed from the list.

The two small-molecules compounds found in this work (i.e., PMPT and CPSQPA) were shown to inhibit the activity of the 3CLpro^SARS– CoV– 2^ with IC_50_ values of 19 and 38 μM in the experimental verification section. Most coronavirus 3CLpro inhibitors have molecular weights in the range of 300–500 g/mol and IC_50_’s in the range of nM to mM (Liu Y. et al., 2020). The molecular weights of the two identified inhibitors were in a similar range with relatively low IC_50_ values. The two newly discovered 3CLpro inhibitors non-covalently bind with the amino acid residues in the S1, S2, and S4 pockets, particularly in catalytically active Cys145 in the S1′ pocket. Additionally, the non-covalent inhibitors are mainly advanced in having weak reversible binding, which could result in avoidance of the off-target risk and toxicity of irreversible inhibitors. These noncovalent inhibitors might be suitable for long-term administration (Liu Y. et al., 2020). Since no toxicity study has been conducted on either of these compounds, future work could test the toxicity of PMPT and CPSQPA.

The pyrimidinetrione group of PMPT served as both a hydrogen bond donor and acceptor, and it is thus presumably an essential functional group. This finding may explain why pyrimidines were reported to be inhibitors of 3CLpro^SARS– CoV– 1^ (Ramajayam et al., 2010). The quinoxalin of CPSQPA may form a hydrogen bond with Gly143, which is close to the active site Cys145 (Supplementary Figure 5). Quinoxalin is a newly found functional group that has not been reported in other known 3CLpro inhibitors. Many quinoline derivatives were tested for their inhibitory effect previously, but the IC_50_ values were generally more than 100 μM (Liu Y. et al., 2020). Quinxalin would be an alternative basic structure to further design 3CLpro inhibitors. Furthermore, the sulfonamide of CPSQPA appears in some known 3CLpro inhibitors, including 5-sulfonyl isatin derivatives that inhibited 3CLpro^SARS– CoV– 1^ in the low micromolar range (Liu et al., 2014). Therefore, pyrimidinetrione and quinoxalin derivatives would be good starting points to find additional 3CLpro inhibitors. These two identified inhibitors have similar features to known inhibitors, and at the same time they provide new basic chemical structures for the further lead optimization.

## Conclusion

In order to inhibit the replication of SARS-CoV-2, an integrated computational and experimental approach was developed in this work to identify potential compounds that inhibit 3CLpro^SARS– CoV– 2^. 288 potential inhibitors of the main protease (3CLpro) of SARS-CoV-2 were identified through virtual screening of half a million compounds from existing databases. Inhibitory activities of the compounds were predicted from a QSAR model developed from existing data for the inhibitors of 3CLpro^SARS– CoV– 1^. Among these potential inhibitors, 71 compounds were further selected for validation via an enzyme activity assay, and 9 compounds showed certain inhibition of 3CLpro. Among these compounds, PMPT and CPSQPA were confirmed by experiments to effectively inhibit the activity of 3CLpro^SARS– CoV– 2^, with IC_50_ values of 19 ± 3 μM and 38 ± 3 μM, respectively. The functional groups pyrimidinetrione and quinoxaline were newly found in 3CLpro inhibitors, thus they are of high interest for lead optimization. In future studies, cellular infection and animal testing could be conducted to validate the efficacy and safety of the two newly identified compounds.

## Data Availability Statement

The original contributions presented in the study are included in the article/Supplementary Material, further inquiries can be directed to the corresponding author/s.

## Author Contributions

TZ, FZ, SH, DK, and ZH: plan of the study and analysis of results. TZ, SH, and ZH: computational. TZ, FZ, DK, and ZH: experiment. TZ and FZ: draft the manuscript. SH, DK, and ZH: draft revision. All authors contributed to the article and approved the submitted version.

## Conflict of Interest

The authors declare that the research was conducted in the absence of any commercial or financial relationships that could be construed as a potential conflict of interest.

## Supplementary Material

Supplementary Table 1 Enzyme activity and IC_50_ data of PMPT and CPSQPA.

Supplementary Table 2 Inhibitory activity evaluation of 71 compounds.

Supplementary Table 3 The data for QSAR modeling and Docking simulation.

## References

[B1] Abian O. Ortega-Alarcon D. Jimenez-Alesanco A. Ceballos-Laita L. Vega S. Reyburn H. T. (2020). Structural stability of SARS-CoV-2 3CLpro and identification of quercetin as an inhibitor by experimental screening. *Int. J. Biol. Macromol.* 164 1693–1703. 10.1016/j.ijbiomac.2020.07.235 32745548 PMC7395220

[B2] Achilonu I. Iwuchukwu E. A. Achilonu O. J. Fernandes M. A. (2020). Targeting the SARS-CoV-2 main protease using FDA-approved Isavuconazonium, a P2-P3 α-ketoamide derivative and Pentagastrin: an in-silico drug discovery approach. *Res. Square* [Preprint]. 10.21203/rs.3.rs-30382/v1 PMC7462840 32920239

[B3] Anand K. Ziebuhr J. Wadhwani P. Mesters J. R. Hilgenfeld R. (2003). Coronavirus main proteinase (3CLpro) structure: basis for design of anti-SARS drugs. *Science* 300 1763–1767. 10.1126/science.1085658 12746549

[B4] Bacha U. Barrila J. Velazquez-Campoy A. Leavitt S. A. Freire E. (2004). Identification of Novel Inhibitors of the SARS Coronavirus Main Protease 3CLpro. *Biochemistry* 43 4906–4912. 10.1021/bi0361766 15109248

[B5] Balouiri M. Sadiki M. Ibnsouda S. K. (2016). Methods for in vitro evaluating antimicrobial activity: a review. *J. Pharmaceut. Anal.* 6 71–79. 10.1016/j.jpha.2015.11.005 29403965 PMC5762448

[B6] Barrila J. Bacha U. Freire E. (2006). Long-range cooperative interactions modulate dimerization in SARS 3CLpro. *Biochemistry* 45 14908–14916. 10.1021/bi0616302 17154528 PMC2570436

[B7] Beigel J. H. Tomashek K. M. Dodd L. E. Mehta A. K. Zingman B. S. Kalil A. C. (2020). Remdesivir for the Treatment of Covid-19 — Final Report. *N. Engl. J. Med.* 383 1813–1826. 32445440 10.1056/NEJMoa2007764 PMC7262788

[B8] CDC (2021a). *Emerging SARS-CoV-2 Variants* [Online]. Available online at: https://www.cdc.gov/coronavirus/2019-ncov/more/science-and-research/scientific-brief-emerging-variants.html [Accessed Jan 3 2021].

[B9] CDC (2021b). *Selected Adverse Events Reported after COVID-19 Vaccination [Online]*. Available at: https://www.cdc.gov/coronavirus/2019-ncov/vaccines/safety/adverse-events.html [Accessed April 6, 2021].

[B10] CSSE (2020). COVID-19 Dashboard by the Center for Systems Science and Engineering (CSSE) at Johns Hopkins University (JHU) [Online]. Available online at: https://coronavirus.jhu.edu/map.html (accessed Decemer 31, 2020).

[B11] De Wilde A. H. Snijder E. J. Kikkert M. Van Hemert M. J. (2018). Host factors in coronavirus replication. *Curr. Top. Microbiol. Immunol.* 419 1–42. 10.1007/82_2017_25 28643204 PMC7119980

[B12] Dhama K. Sharun K. Tiwari R. Dadar M. Malik Y. S. Singh K. P. (2020). COVID-19, an emerging coronavirus infection: advances and prospects in designing and developing vaccines, immunotherapeutics, and therapeutics. *Hum. Vaccin. Immunother*, 16, 1232–1238. 10.1080/21645515.2020.1735227 32186952 PMC7103671

[B13] Fu L. Ye F. Feng Y. Yu F. Wang Q. Wu Y. (2020). Both Boceprevir and GC376 efficaciously inhibit SARS-CoV-2 by targeting its main protease. *Nat. Commun.* 11:4417. 10.1038/s41467-020-18233-x PMC7474075 32887884

[B14] Grein J. Ohmagari N. Shin D. Diaz G. Asperges E. Castagna A. (2020). Compassionate Use of Remdesivir for Patients with Severe Covid-19. *N. Engl. J. Med.* 382 2327–2336. 32275812 10.1056/NEJMoa2007016 PMC7169476

[B15] Hilgenfeld R. Peiris M. (2013). From SARS to MERS: 10 years of research on highly pathogenic human coronaviruses. *Antiv. Res.* 100 286–295. 10.1016/j.antiviral.2013.08.015 24012996 PMC7113673

[B16] Huang C. Wang Y. Li X. Ren L. Zhao J. Hu Y. (2020). Clinical features of patients infected with 2019 novel coronavirus in Wuhan, China. *Lancet* 395 497–506. 31986264 10.1016/S0140-6736(20)30183-5 PMC7159299

[B17] Jacobs J. Grum-Tokars V. Zhou Y. Turlington M. Saldanha S. A. Chase P. (2013). Discovery, synthesis, and structure-based optimization of a series of N-(tert-Butyl)-2-(N-arylamido)-2-(pyridin-3-yl) Acetamides (ML188) as Potent noncovalent small molecule inhibitors of the severe acute respiratory syndrome coronavirus (SARS-CoV) 3CL protease. *J. Med. Chem.* 56 534–546. 10.1021/jm301580n 23231439 PMC3569073

[B18] Jin Z. Du X. Xu Y. Deng Y. Liu M. Zhao Y. (2020). Structure of Mpro from SARS-CoV-2 and discovery of its inhibitors. *Nature* 582 289–293. 32272481 10.1038/s41586-020-2223-y

[B19] Kitchen D. B. Decornez H. Furr J. R. Bajorath J. (2004). Docking and scoring in virtual screening for drug discovery: methods and applications. *Nat. Rev. Drug Discov.* 3 935–949. 10.1038/nrd1549 15520816

[B20] Klemm T. Ebert G. Calleja D. J. Allison C. C. Richardson L. W. Bernardini J. P. (2020). Mechanism and inhibition of the papain-like protease, PLpro, of SARS-CoV-2. *EMBO J.* 39:e106275. 10.15252/embj.2020106275 PMC7461020 32845033

[B21] Kong W.-H. Li Y. Peng M.-W. Kong D.-G. Yang X.-B. Wang L. (2020). SARS-CoV-2 detection in patients with influenza-like illness. *Nat. Microbiol.* 5 675–678. 10.1038/s41564-020-0713-1 32265517

[B22] Lai C.-C. Shih T.-P. Ko W.-C. Tang H.-J. Hsueh P.-R. (2020). Severe acute respiratory syndrome coronavirus 2 (SARS-CoV-2) and coronavirus disease-2019 (COVID-19): The epidemic and the challenges. *Int. J. Antimicrob. Agents* 55:105924. 10.1016/j.ijantimicag.2020.105924 PMC7127800 32081636

[B23] Li G. De Clercq E. (2020). *Therapeutic Options for the 2019 Novel Coronavirus (2019-nCoV).* Berlin: Nature Publishing Group. 10.1038/d41573-020-00016-0 32127666

[B24] Lipinski C. A. Lombardo F. Dominy B. W. Feeney P. J. (1997). Experimental and computational approaches to estimate solubility and permeability in drug discovery and development settings. *Adv. Drug Deliv. Rev.* 23 3–25. 10.1016/s0169-409x(96)00423-1 11259830

[B25] Liu C. Zhou Q. Li Y. Garner L. V. Watkins S. P. Carter L. J. (2020). Research and development on therapeutic agents and vaccines for COVID-19 and related human coronavirus diseases. *ACS Cent. Sci.* 6 315–331. 10.1021/acscentsci.0c00272 32226821 PMC10467574

[B26] Liu W. Zhu H. M. Niu G. J. Shi E. Z. Chen J. Sun B. (2014). Synthesis, modification and docking studies of 5-sulfonyl isatin derivatives as SARS-CoV 3C-like protease inhibitors. *Bioorg. Med. Chem.* 22 292–302. 10.1016/j.bmc.2013.11.028 24316352 PMC7111328

[B27] Liu Y. Liang C. Xin L. Ren X. Tian L. Ju X. (2020). The development of coronavirus 3C-Like protease (3CL(pro)) inhibitors from 2010 to 2020. *Eur. J. Med. Chem.* 206:112711. 10.1016/j.ejmech.2020.112711 32810751 PMC7409838

[B28] Nantasenamat C. Isarankura-Na-Ayudhya C. Naenna T. Prachayasittikul V. (2009). A practical overview of quantitative structure-activity relationship. *EXCLI J.* 8 74–88.

[B29] Neves M. a. C. Totrov M. Abagyan R. (2012). Docking and scoring with ICM: the benchmarking results and strategies for improvement. *J. Comput. Aided Mol. Design* 26 675–686. 10.1007/s10822-012-9547-0 22569591 PMC3398187

[B30] Patridge E. Gareiss P. Kinch M. S. Hoyer D. (2016). An analysis of FDA-approved drugs: natural products and their derivatives. *Drug Discov. Today* 21 204–207. 10.1016/j.drudis.2015.01.009 25617672

[B31] Perlman S. Netland J. (2009). Coronaviruses post-SARS: update on replication and pathogenesis. *Nat. Rev. Microbiol.* 7 439–450. 10.1038/nrmicro2147 19430490 PMC2830095

[B32] Pillaiyar T. Manickam M. Namasivayam V. Hayashi Y. Jung S. H. (2016). An overview of severe acute respiratory syndrome-coronavirus (SARS-CoV) 3CL protease inhibitors: peptidomimetics and small molecule chemotherapy. *J. Med. Chem.* 59 6595–6628. 10.1021/acs.jmedchem.5b01461 26878082 PMC7075650

[B33] Ramajayam R. Tan K. P. Liu H. G. Liang P. H. (2010). Synthesis, docking studies, and evaluation of pyrimidines as inhibitors of SARS-CoV 3CL protease. *Bioorg. Med. Chem. Lett.* 20 3569–3572. 10.1016/j.bmcl.2010.04.118 20494577 PMC7126861

[B34] Ratia K. Pegan S. Takayama J. Sleeman K. Coughlin M. Baliji S. (2008). A noncovalent class of papain-like protease/deubiquitinase inhibitors blocks SARS virus replication. *Proc. Natl. Acad. Sci. U.S.A.* 105 16119–16124. 10.1073/pnas.0805240105 18852458 PMC2571001

[B35] Roy S. Kumar A. Baig M. H. Masaøík M. Provazník I. (2015). Virtual screening, ADMET profiling, molecular docking and dynamics approaches to search for potent selective natural molecules based inhibitors against metallothionein-III to study Alzheimer’s disease. *Methods* 83 105–110. 10.1016/j.ymeth.2015.04.021 25920949

[B36] Shi J. Sivaraman J. Song J. (2008). Mechanism for controlling the dimer-monomer switch and coupling dimerization to catalysis of the severe acute respiratory syndrome coronavirus 3C-like protease. *J. Virol.* 82 4620–4629. 10.1128/jvi.02680-07 18305031 PMC2293028

[B37] Sirois S. Wei D.-Q. Du Q. Chou K.-C. (2004). Virtual screening for SARS-CoV protease based on KZ7088 pharmacophore points. *J. Chem. Inform. Comput. Sci.* 44 1111–1122. 10.1021/ci034270n 15154780

[B38] Stertz S. Reichelt M. Spiegel M. Kuri T. Martínez-Sobrido L. García-Sastre A. (2007). The intracellular sites of early replication and budding of SARS-coronavirus. *Virology* 361 304–315. 10.1016/j.virol.2006.11.027 17210170 PMC7103305

[B39] Totrov M. (2008). Atomic property fields: generalized 3D pharmacophoric potential for automated ligand superposition, pharmacophore elucidation and 3D QSAR. *Chem. Biol. Drug Design* 71 15–27. 10.1111/j.1747-0285.2007.00605.x 18069986

[B40] Totrov M. (2011). Ligand binding site superposition and comparison based on Atomic Property Fields: identification of distant homologues, convergent evolution and PDB-wide clustering of binding sites. *BMC Bioinform.* 12(Suppl. 1):S35–S35. 10.1186/1471-2105-12-S1-S35 PMC3044291 21342566

[B41] Turlington M. Chun A. Tomar S. Eggler A. Grum-Tokars V. Jacobs J. (2013). Discovery of N-(benzo[1,2,3]triazol-1-yl)-N-(benzyl)acetamido)phenyl) carboxamides as severe acute respiratory syndrome coronavirus (SARS-CoV) 3CLpro inhibitors: identification of ML300 and noncovalent nanomolar inhibitors with an induced-fit binding. *Bioorg. Med. Chem. Lett.* 23 6172–6177. 10.1016/j.bmcl.2013.08.112 24080461 PMC3878165

[B42] Xu Z. Shi L. Wang Y. Zhang J. Huang L. Zhang C. (2020). Pathological findings of COVID-19 associated with acute respiratory distress syndrome. *Lancet Respirat. Med.* 8, 420–422. 10.1016/S2213-2600(20)30076-X 32085846 PMC7164771

[B43] Zhang F. Zhai T. Haider S. Liu Y. Huang Z. J. (2020). Synergistic effect of chlorogenic acid and caffeic acid with fosfomycin on growth inhibition of a resistant listeria monocytogenes strain. *ACS Omega* 5, 7537–7544. 10.1021/acsomega.0c00352 32280897 PMC7144146

[B44] Zhang L. Lin D. Kusov Y. Nian Y. Ma Q. Wang J. (2020). α-ketoamides as broad-spectrum inhibitors of coronavirus and enterovirus replication: structure-based design, synthesis, and activity assessment. *J. Med. Chem.* 63 4562–4578. 10.1021/acs.jmedchem.9b01828 32045235

