# Supplementary material for: An Integrated Computational and Experimental Approach to Identifying Inhibitors for SARS-CoV-2 3CL Protease
Source: Front Mol Biosci. 2021 May 17;8:661424. doi: 10.3389/fmolb.2021.661424 (PMC8166273; doi:10.3389/fmolb.2021.661424)
Supplement: Supplementary file 1 [file Image_1.pdf]

Supplemental figures

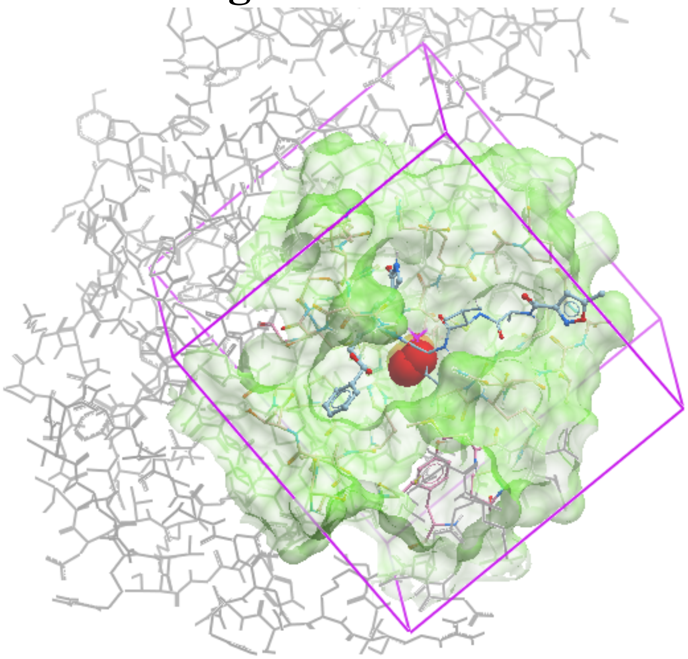

Supplement Figure 1. Docking box of the receptor 6LU7

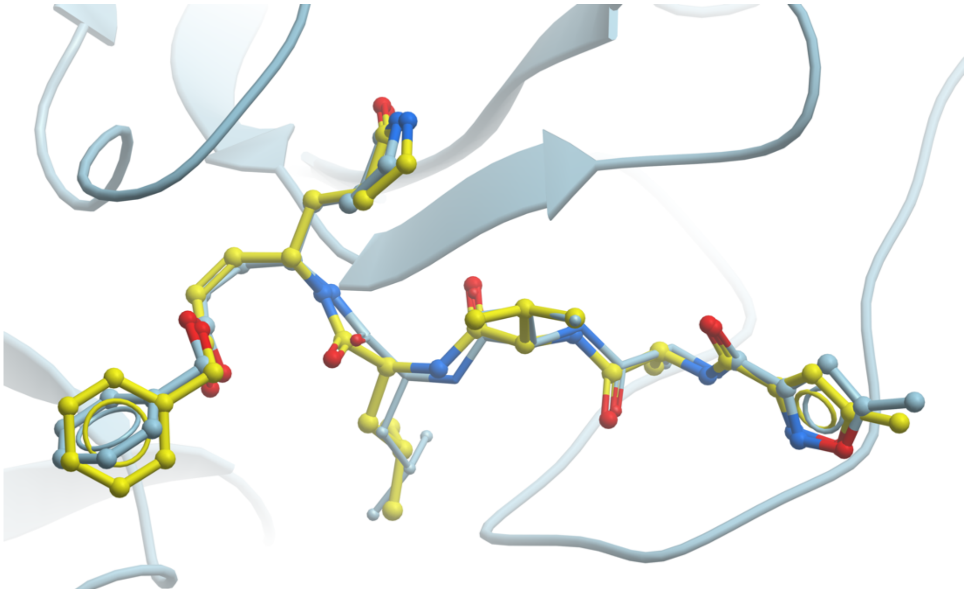

Supplement Figure 2. the redocking pose of N3 in structure 6LU7. Crystal ligand was colored in skyblue, and the docking pose was yellow. The docking conformation returned a score of ΔG = -29.02 kcal/mol and an RMSD of 0.6 Å relative to the original.

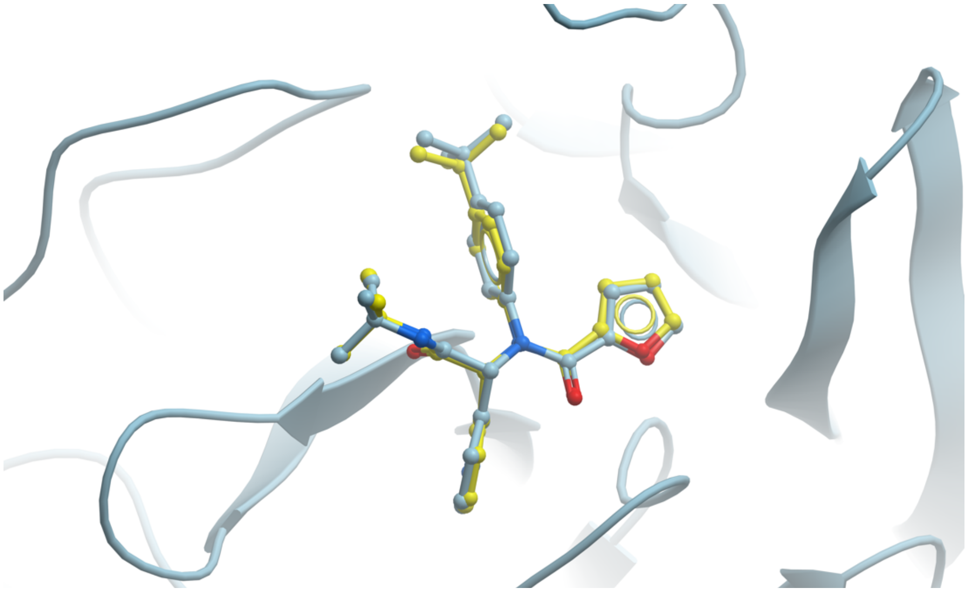

Supplement Figure 3. The redocking pose of ML188 in structure 7L0D. The redocked Crystal ligand was colored in skyblue, and the docking pose was yellow. The docking conformation returned a score of ΔG = -28.02 kcal/mol and an RMSD of 0.86 Å relative to the original.

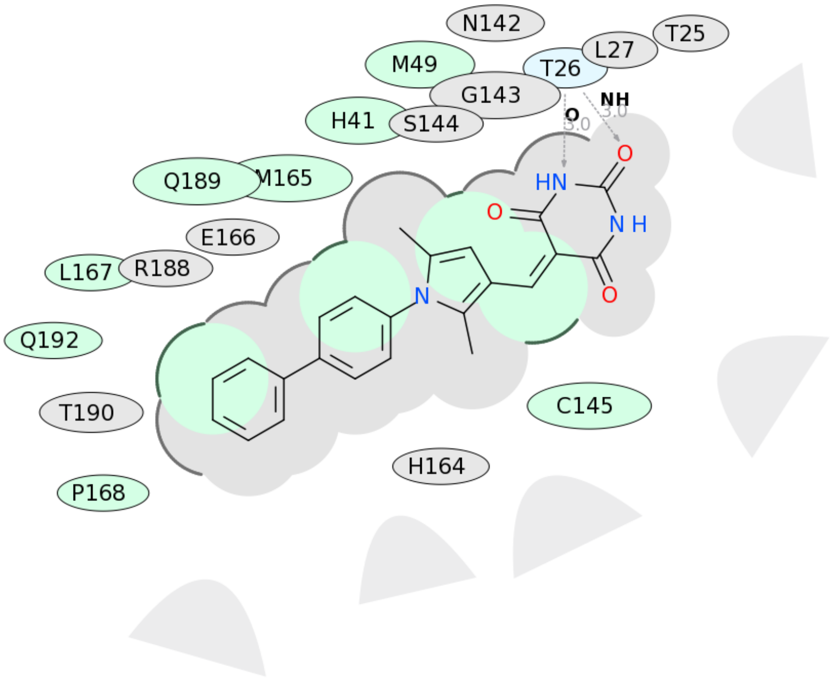

Supplement Figure 4. The 2D illustration of the interaction between PMPT and 3CLpro. The image is annotated with hydrogen bonds and interacting residues. The residue interaction surface and proximity are represented by the size of the residue label and distance, respectively. Grey

parabolas and broken thick lines indicate solvent accessible regions and the ligand is shaded by property. Here are the meanings of colors, lines, and sizes shown in the 2D interaction diagram: 1) Green shading represents hydrophobic region; 2) Blue shading represents hydrogen bond acceptor; 3) White dashed arrows represent hydrogen bonds; 4) Grey parabolas represent accessible surface for large areas; 5) Broken thick line around ligand shape indicates accessible surface; 6) Size of residue ellipse represents the strength of the contact; 7) 2D distance between residue label and ligand represents proximity; 8) Covalently bound residue represented with thick black line around ellipse.

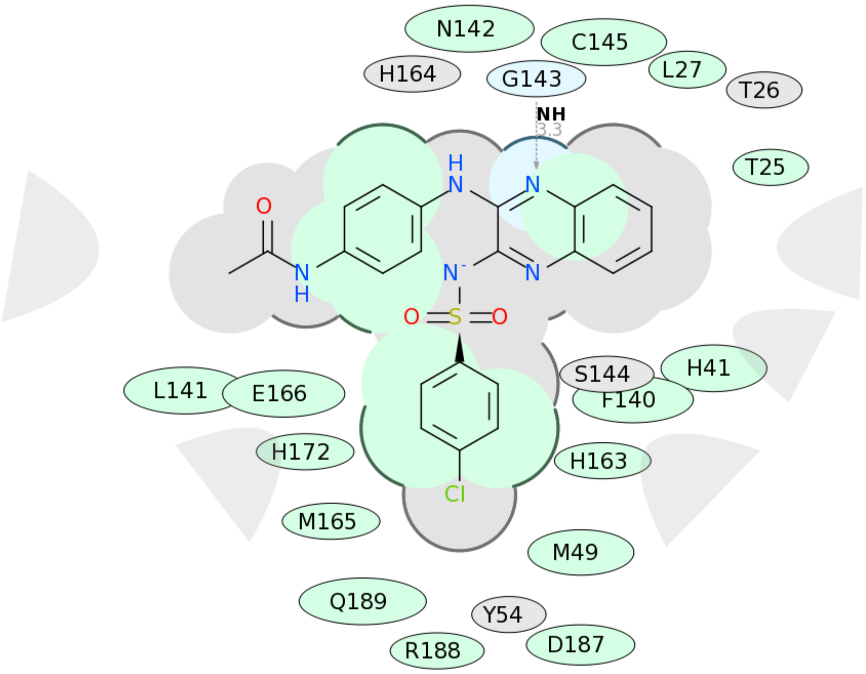

Supplement Figure 5. The 2D illustration of the interaction between of CPSQPA and 3CLpro. The image is annotated with hydrogen bonds and interacting residues. The residue interaction surface and proximity are represented by the size of the residue label and distance, respectively. Grey parabolas and broken thick lines indicate solvent accessible regions and the ligand is shaded by property. Here are the meanings of colors, lines, sizes shown in the 2D interaction diagram: 1) Green shading represents hydrophobic region; 2) Blue shading represents hydrogen bond acceptor; 3) White dashed arrows represents hydrogen bonds; 4) Grey parabolas represents accessible surface for large areas; 5) Broken thick line around ligand shape indicates accessible surface; 6) Size of residue ellipse represents the strength of the contact; 7) 2D distance between residue label and ligand represents proximity; 8) Covalently bound residue represented with thick black line around ellipse.

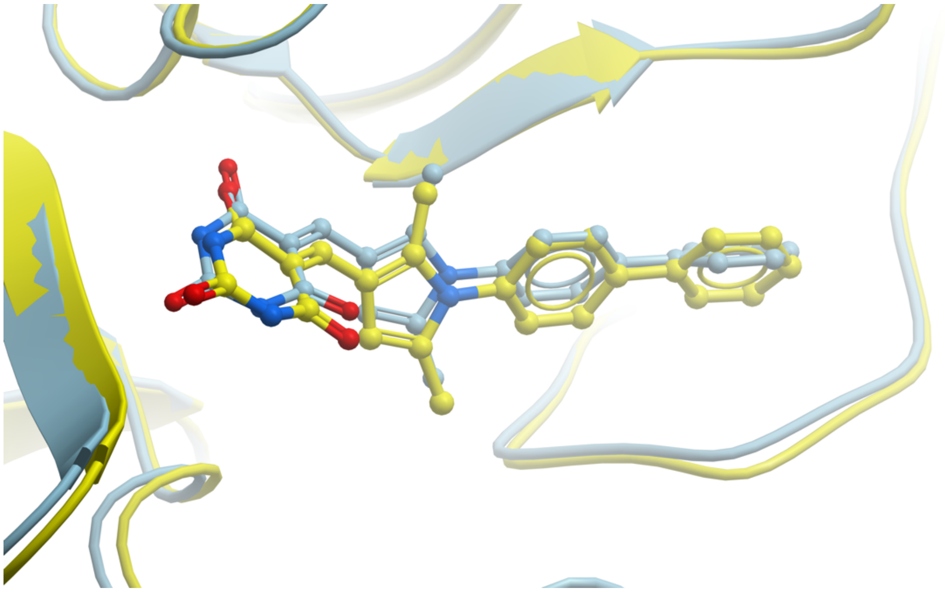

Supplement Figure 6. Docking poses of PMPT in two structures 6LU7 (blue) and 7L0D (yellow).

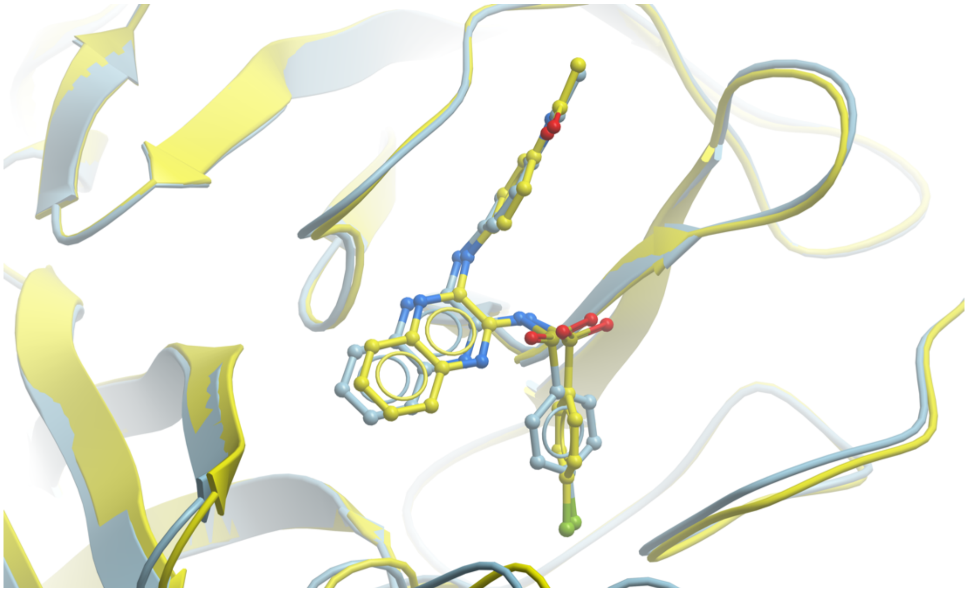

Supplement Figure 7. Docking poses of CPSQPA in two structures 6LU7 (blue) and 7L0D (yellow).
